# Rapid Microwave Polymerization of Porous Nanocomposites with Piezoresistive Sensing Function

**DOI:** 10.3390/nano10020233

**Published:** 2020-01-29

**Authors:** Blake Herren, Mohammad Charara, Mrinal C. Saha, M. Cengiz Altan, Yingtao Liu

**Affiliations:** School of Aerospace and Mechanical Engineering, University of Oklahoma, Norman, OK 73019, USA; blake.herren@ou.edu (B.H.); mcharara@ou.edu (M.C.); msaha@ou.edu (M.C.S.); altan@ou.edu (M.C.A.)

**Keywords:** microwave irradiation, nanocomposite, elastomer, polydimethylsiloxane, carbon nanotubes, piezoresistive sensor, microstructures

## Abstract

In this paper, polydimethylsiloxane (PDMS) and multi-walled carbon nanotube (MWCNT) nanocomposites with piezoresistive sensing function were fabricated using microwave irradiation. The effects of precuring time on the mechanical and electrical properties of nanocomposites were investigated. The increased viscosity and possible nanofiller re-agglomeration during the precuring process caused decreased microwave absorption, resulting in extended curing times, and decreased porosity and electrical conductivity in the cured nanocomposites. The porosity generated during the microwave-curing process was investigated with a scanning electron microscope (SEM) and density measurements. Increased loadings of MWCNTs resulted in shortened curing times and an increased number of small well-dispersed closed-cell pores. The mechanical properties of the synthesized nanocomposites including stress–strain behaviors and Young’s Modulus were examined. Experimental results demonstrated that the synthesized nanocomposites with 2.5 wt. % MWCNTs achieved the highest piezoresistive sensitivity with an average gauge factor of 7.9 at 10% applied strain. The piezoresistive responses of these nanocomposites were characterized under compressive loads at various maximum strains, loading rates, and under viscoelastic stress relaxation conditions. The 2.5 wt. % nanocomposite was successfully used in an application as a skin-attachable compression sensor for human motion detection including squeezing a golf ball.

## 1. Introduction

The development of multi-functional porous nanocomposites capable of large deformation and force sensing with high sensitivity, good reliability, and biocompatibility are of considerable interest in wearable and flexible electronics. Recent studies have shown that the introduction of highly conductive nanoparticles to the porous surfaces or microstructures in nanocomposites can significantly improve the sensitivity and linear sensing range [1]. More recently, the control of microstructures in porous nanocomposites was regarded as an attractive approach to increase the sensing range and improve the sensor performance [2,3]. Various pore sizes and porosities have been investigated to optimize the properties and sensing functions in nanocomposites.

The most effective porous nanocomposites with high load sensing capabilities consist of an elastomeric polymer matrix and highly conductive nanoparticles. Flexible and stretchable elastomers, such as polydimethylsiloxane (PDMS), have been employed as the matrix and broadly used in the development of porous nanocomposites. Due to its high flexibility, large Poisson ratio, transparency, and biocompatibility, PDMS has been widely reported in literature for the development of flexible nanocomposites [4,5,6]. These attributes have led to its use in the development of highly flexible and stretchable nanocomposite strain sensors and pressure sensors for several different applications [7,8]. To improve the electrical conductivity in the developed nanocomposites, various nanoparticles, such as carbon black [9,10,11], carbon nanofibers [12,13], multi-walled carbon nanotubes (MWCNTs) [14,15,16,17,18], and graphene [19,20], have been studied to create porous nanocomposites. Of these nanofillers, MWCNTs have received considerable interest due to their ability to simultaneously improve the thermal, mechanical, and electrical properties of nanocomposites [21]. In addition, good sensitivity and high gauge factors have been achieved [17,22]. Many techniques have been used to enhance the dispersion of MWCNTs within a polymer including shear mixing, ultrasonication, and MWCNT functionalization to provide the optimal properties to the nanocomposites [23]. Direct extrusion based 3D printing methods have been reported to control the alignment of nanoparticles in polymer nanocomposites for microstructural and property optimization [24,25].

A wide variety of innovative manufacturing methods have been developed to improve properties and functionality of nanocomposites, and to increase fabrication efficiency. Advanced manufacturing techniques, such as 3D printing [7,26,27] and microwave irradiation [28,29,30], have been used to fabricate MWCNT-based nanocomposites with complex geometries and controlled microstructures to improve the versatility and efficiency of the manufacturing process. Using these techniques and many other manufacturing methods, researchers have made remarkable progress in the development of MWCNT doped epoxies [31], composite laminates [32], and flexible conductive nanocomposites [33].

Microwave irradiation is efficient in curing thermoset resins as it utilizes interactions at the molecular level to heat materials from inside the material itself, rather than heating an environment and allowing the heat to propagate from the outside. This heating process leads to significantly reduced curing times due to more efficient and localized energy delivery [34]. The ability of microwave irradiation to quickly cure a thermoset resin is substantially enhanced with the addition of carbonaceous nanofillers. These carbon-based nanoparticles have high microwave absorption, therefore when dispersed within a prepolymer, they can provide localized heat throughout the uncured thermoset to rapidly cure the material [35]. Carbon nanofillers, such as MWCNTs, have been implemented recently to rapidly microwave-cure epoxies and experimental results have shown that MWCNT doped thermosets cured in a microwave have comparable or improved properties over those cured in an oven [30,36,37,38]. Rangari et al. demonstrated a reduction in curing time from 8 h in an oven to 10 min in a microwave, in addition to a 17% increase in strength of microwave cured EPON862 resin with 0.2 wt. % MWCNT loading over thermally-cured neat epoxy [30]. Fotiou et al. reported savings in energy of at least 40% for MWCNTs doped epoxy cured in a microwave in comparison to a conventional oven [36]. Due to the significantly reduced curing times and improved energy efficiency over thermal-curing, it is important that microwave-curing of thermosets containing dispersed carbonaceous nanofillers be fully explored.

Advanced manufacturing techniques have been developed to fabricate porous PDMS structures to enhance the piezoresistive function of the nanocomposite, including sugar templating, a process that uses a sugar cube as a sacrificial porogen. Although effective, this method is time-consuming, difficult to scale-up, and complicated. Recently, methods have been developed to produce porous PDMS structures using microwave irradiation and sacrificial solvents. Notably, Jang et al. used a facile microwave-curing technique to fabricate porous PDMS foam for triboelectric nanogenerators with the utilization of sacrificial solvents [39]. To the best of our knowledge, using sacrificial solvents and microwave-curing to rapidly induce porosity in PDMS nanocomposites with piezoresistive function has not been explored before. In our previous work, we showed that the electrical conductivity of PDMS nanocomposites containing dispersed MWCNTs could be enhanced up to 142.8% by microwave-curing the nanocomposite with optimal commercial microwave settings [37]. Rapid microwave-curing improved the MWCNT dispersion by reducing settlement effect apparent during thermal-curing and possibly aligned the MWCNTs, which further increased the conductivity of the nanocomposite.

In this study, one-step microwave irradiation was used to fabricate porous conductive nanocomposites through simultaneous rapid curing and residual solvent evaporation. One-step microwave-curing was applied to PDMS containing dispersed MWCNTs and residual tetrahydrofuran (THF) to demonstrate the simplicity of fabrication and to investigate the pores produced in the nanocomposites as a result of high internal temperature build-up and subsequent solvent evaporation. It has been reported in the literature that dielectric loss factor and consequently microwave absorption of thermosets decrease due to partial curing [36]. This phenomenon was investigated in this study by precuring the nanocomposite prepolymer before microwave-curing to explore the effects on the curing process, porosity, and electrical conductivity. Additionally, nanocomposites with different loadings of MWCNT were fabricated to compare their resulting mechanical properties and piezoresistive sensing functions. A scanning electron microscope (SEM) was used to qualify the porosity and demonstrate the quality of MWCNT dispersion within the nanocomposites. The nanocomposite with the best piezoresistive performance was further investigated to fully characterize the piezoresistive response of the porous compression sensor.

## 2. Materials and Methods

### 2.1. Materials

Unless otherwise stated, all materials were used as received. MWCNTs (diameter 50–80 nm and aspect ratio >100) were purchased from Sigma Aldrich (St. Louis, MO, USA). The SYLGARD 184 PDMS was purchased from Dow Corning (Midland, MI, USA). SYLGARD 184 is a two-part PDMS, consisting of the base elastomer (part A) and curing agent (part B). Tetrahydrofuran (THF) was used as the solvent for MWCNT dispersion and was purchased from Sigma Aldrich.

### 2.2. Nanocomposite Resin Preparation

**Figure 1:**
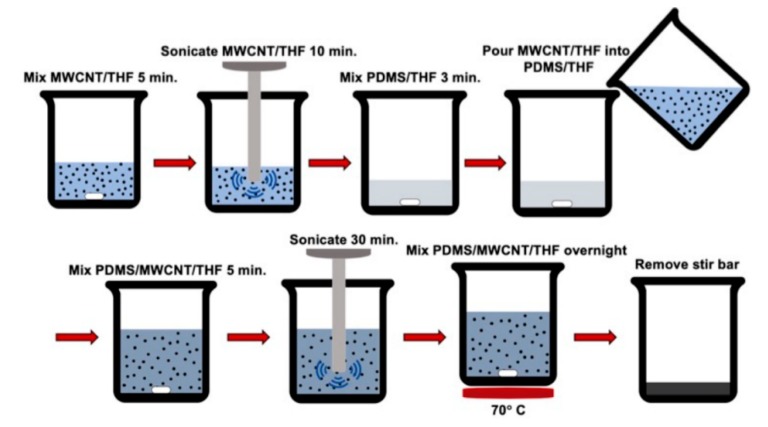
Schematic of the nanoparticle dispersion process used to fabricate the nanocomposite resin.

A solvent-based ultrasonication method was used to disperse MWCNTs in the PDMS base elastomer. MWCNTs were first kept in a vacuum oven overnight at 110 °C to remove any trace of moisture before dispersion. A selected amount of MWCNTs was added to 30 mL of THF and ultrasonicated with a 750-watt probe sonicator for 10 min to disperse the nanotubes within the THF. The probe sonicator was pulsed on for 5 s and off for 2 s to decrease the possibility of MWCNTs being damaged during the dispersion process. Concurrently, PDMS part A was added to 20 mL of THF and mixed at 350 rpm with a magnetic stir bar for 3 min to reduce its viscosity. Then, the nanoparticle suspension was added to the PDMS part A solution and sonicated for 30 min. The nanocomposite mixture was placed on a hot plate at 70 °C and mixed at 350 rpm overnight to evaporate the THF. Lastly, an appropriate amount of curing agent was added to the mixture and well-mixed before microwave-curing. A schematic of this process is shown in Figure 1. Nanocomposite resins with 1.0 wt. %, 1.5 wt. %, 2.0 wt. %, and 2.5 wt. % MWCNTs were prepared using the method described.

### 2.3. Piezoresistive Sensor Fabrication

The nanocomposite resin was mixed with the curing agent at the manufacturer recommended 10:1 ratio (part A: part B) for 5 min to form the nanocomposite prepolymer. The material was then loaded into a 3 mL syringe and extruded into a cylindrical glass mold (diameter = 11 mm, height = 9 mm). The top surface was smoothed before placing in the center of the microwave. A commercial General Electric 1200-watt microwave (GE, Boston, MA, USA) was used at 50% power for the microwave-curing procedure. One-step microwave exposure was used for simplicity, time-efficiency, potentially improved MWCNT dispersion and alignment, and to initiate the maximum porosity due to rapid temperature build-up and solvent evaporation. Four porous types of nanocomposites with 1.0 wt. %, 1.5 wt. %, 2.0 wt. %, and 2.5 wt. % MWCNTs were cured using microwave irradiation. The curing time of these four types of nanocomposites was 25, 25, 17, and 15 s, respectively.

### 2.4. Rheology

Rheology tests were performed to determine the viscoelastic properties at various loadings of the uncured nanocomposites. The uncured nanocomposites with 1.0 wt. %, 1.5 wt. %, 2.0 wt. %, and 2.5 wt. % MWCNTs were tested using a TA Instruments Discovery HR-2 rheometer. The shear rate was increased slowly from 1 s-1 to 1500 s-1 during each test to ensure an accurate measurement of viscosity. A cone fixture with a 40 mm diameter and a 2o cone angle was used with a test gap of 53 μm in all the rheology experiments.

### 2.5. Partial Precuring Study

To study the effects of precuring the nanocomposite prepolymer on the microwave radiation process, resulting electrical conductivity of the cured nanocomposite, and pore morphology, samples with 1.0 wt. % loading were prepared after precuring the resin at room temperature at chosen time intervals after adding the curing agent. The precuring times tested were 0 min, 30 min, 1 h, 2 h, 4 h, 6 h, 12 h, and 24 h. Four different batches of the nanocomposite resin were prepared to make four different samples at each time interval. Since PDMS’s curing time at room temperature (25 °C) recommended by the vendor is at least 48 h [40], all the studied samples in this section were partially cured before microwave radiation.

### 2.6. SEM Investigation

An SEM was used to characterize the porosity and dispersion state within the fabricated nanocomposites with various precuring times and MWCNT concentrations. The microstructures of the nanocomposite samples that underwent precuring were imaged first to understand the effect of precuring on the porosity of the microwave-cured nanocomposites with a MWCNT concentration of 1.0 wt. %. In addition, images were obtained to evaluate the differences in porosity due to different nanofiller loadings in the prepared nanocomposites. A sample with the highest MWCNT concentration of 2.5 wt. % was imaged to assess the nanoparticle dispersion within the nanocomposite as this sample was most likely to have agglomerates or signs of inadequate dispersion. All samples imaged were sputter-coated before being imaged to minimize potential charging during the imaging process.

### 2.7. Density and Conductivity Measurements

Density and conductivity of the microwave-cured nanocomposites were investigated to understand the effects of precuring and MWCNT concentrations on the formation of voids and properties of the fabricated samples. The density of each cured nanocomposite was calculated using the measured mass and volume. The electrical conductivity of each nanocomposite was measured using the two-probe method by placing the sample between two copper electrodes and applying a 2 N load and waiting for the measured resistances to adequately stabilize before averaging the resistance data over one minute.

### 2.8. Mechanical Characterization

To characterize the mechanical behavior and piezoresistive function of each nanocomposite, cyclic compression tests were performed with an Instron 3345 single column mechanical testing machine. A sample was placed between two copper plates that were soldered to wires and connected to an Agilent 34401a multimeter to measure the resistance of the material throughout each test. Every test was performed with a preload of 2 N to ensure complete contact between the nanocomposite sensors and the copper plates. Mechanical tests were conducted by applying 40% compressive strain to the flexible nanocomposite for 5 cycles at 2 mm/min.

### 2.9. Piezoresistive Characterization

To determine the baseline piezoresistive sensing capability of each sample, 20 cycles of compressive loading were performed at 2 mm/min and 10% maximum strain while recording electrical resistance data. The nanocomposites with the loading that demonstrated the highest sensitivity in the baseline test (2.5 wt. %) were chosen for additional piezoresistivity characterization. First, cyclic tests were performed at maximum compressive strains of 5%, 10%, 20%, and 40% at a constant crosshead speed of 2 mm/min for 15 cycles to characterize the piezoresistive response of the compression sensors in a large strain range. Next, cyclic loading tests were performed at crosshead speed of 2 mm/min, 5 mm/min, 10 mm/min, and 20 mm/min up to a constant 10% maximum strain to explore the effect of loading rate on the piezoresistive response of the nanocomposites. Finally, the stress and piezoresistive stress relaxation behavior of the elastomeric nanocomposite was characterized by holding the 2.5 wt. % sample at 10% compressive strain for 6 h while recording resistance and stress data.

### 2.10. Sensing Application

To demonstrate the potential for these piezoresistive nanocomposites to be used as flexible compression sensors in a number of applications, a 2.5 wt. % sample was placed between a golf ball and the pointer finger while squeezing was applied. This test included slow, medium, and fast squeezing rates, in addition to step squeezing where 5 s holds were applied at maximum and minimum strain.

## 3. Results and Discussions

### 3.1. Rheology

Rheology tests were performed on the nanocomposite prepolymer to determine the reinforcement effects of MWCNT loadings between 1.0 wt. % and 2.5 wt. %. The viscosity of the nanocomposite prepolymer contributed significantly to the formation of the pores during the microwave-curing procedure. Therefore, it was necessary to investigate the viscosities of the uncured resin at the MWCNT loadings explored in this study.

**Figure 2:**
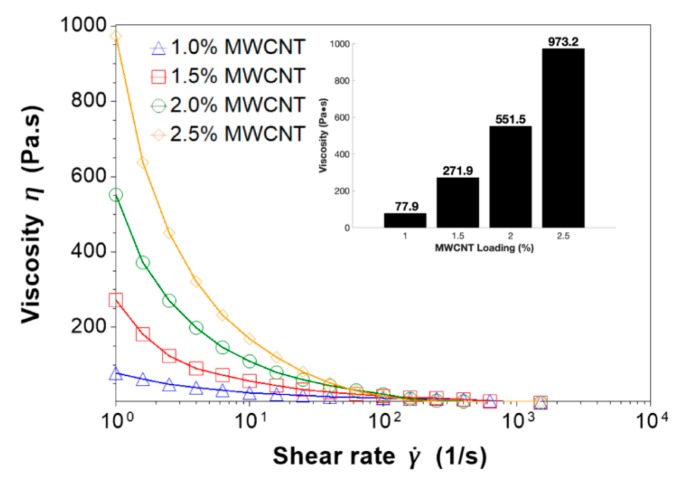
Viscosities of the nanocomposite prepolymer with multi-walled carbon nanotube (MWCNT) concentrations of 1.0 wt. %, 1.5 wt. %, 2.0 wt. %, and 2.5 wt. %.

The measured viscosities of the nanocomposite prepolymer during the rheology tests are shown in Figure 2. The results validated that as the loading of MWCNT increased, the viscosity of the nanocomposite prepolymer increased significantly. This effect was attributed to the impressive reinforcing capabilities of MWCNTs when evenly dispersed in the elastomeric matrix. As the shear rate increased during the experiments, the measured viscosities decreased and eventually converged to a viscosity between 0.1 and 2 Pa⋅s at a shear rate of 1500 s^−1^. This shear thinning effect can be explained by the viscous behavior of the viscoelastic resin dominating at high shear rates. In this paper, the material was not subjected to high shear rates during the microwave-curing procedure; therefore, the relevant viscosity for this study was chosen at the lowest shear rate of 1 s^−1^. The inset in Figure 2 shows that at shear rate of 1 s^−1^ the measured viscosities of the 1.0 wt. %, 1.5 wt. %, 2.0 wt. %, and 2.5 wt. % nanocomposite prepolymers were 77.9 Pa⋅s, 271.9 Pa⋅s, 551.5 Pa⋅s, and 973.2 Pa⋅s, respectively.

### 3.2. Partial Precuring Before Microwave Cure

During preliminary tests to fabricate porous nanocomposites, we observed that the curing times and porosity in the samples produced would vary considerably depending on the amount of time that elapsed after adding the curing agent and before microwave-curing. The optimal time after adding the curing agent was chosen to satisfy three main goals including time efficiency, utilizing pores for improving piezoresistive behavior, and consistent sensor morphology. Therefore, this investigation was necessary to determine the best time to microwave-cure the nanocomposite resin after the curing agent was introduced.

**Figure 3:**
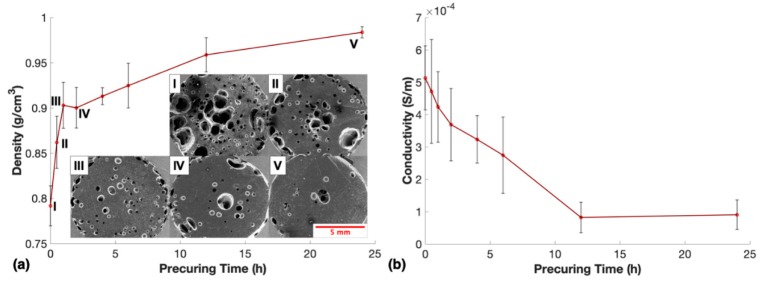
(**a**) Change of density and (**b**) electrical conductivity due to precuring time at room temperature for nanocomposites with 1.0 wt. % MWCNTs and scanning electron microscope (SEM) images of porosity in samples when cured: (i) immediately, (ii) with 30-min precuring, (iii) with 1-h precuring, (iv) with 2-h precuring, and (v) with 24-h precuring.

The effects of these observations were investigated by allowing the nanocomposite prepolymer to partially cure at room temperature before microwave-curing. The lowest MWCNT loading of 1.0 wt. % was chosen for this study as the initial viscosity of this material was significantly lower than 1.5 wt. %, 2.0 wt. %, and 2.5 wt. % due to reinforcing effects endowed by the MWCNTs. Additionally, based on initial observations, the nanocomposite prepolymer with lower MWCNT loadings cured faster at room temperature, which made the change in viscosity as a result of the precuring most apparent for the 1.0 wt. % resin. As precuring time increased, the nanocomposite prepolymer exhibited a progressive increase in time to fully cure via microwave irradiation from 25 s up to 50 s, a decrease of material expansion beyond the top edge of the mold, and a progressive decrease in the overall porosity within the fabricated nanocomposites. Figure 3a shows the change in density due to precuring time in addition to SEM images that illustrated the porosity apparent in the nanocomposites.

The variation of the measured density indicated that the precuring time was an important parameter to consider when using one-step microwave irradiation to fabricate these porous nanocomposites. To take advantage of the closed-cell porosity to improve piezoresistive performance, ideally, the pores would be consistently small and evenly dispersed throughout the sensor. This is not the case for samples cured after less than one hour of precuring (Figure 3aI, II), as the SEM images show very large amorphous pores unevenly distributed throughout the nanocomposite. Large pores that were not evenly distributed caused large variation in piezoresistive and mechanical behavior during cyclic compressive loading tests. These large pores did not collapse fully until a significant strain was applied to the sensor, which resulted in low piezoresistive sensitivity at small strains and inconsistence in resistance change at larger strains. Therefore, a larger number of smaller well-distributed pores were desirable for the enhanced piezoresistive performance of the nanocomposites as the complete collapse of smaller pores occurred at lower strains and the resulting change of conductive networks were more uniform and repeatable. To determine what impact the precuring time before microwave exposure had on the electrical properties of the material, conductivity tests were performed on all samples fabricated for this study, as shown in Figure 3b.

Decreased porosity due to increased precuring time led to an increase in the average cross-sectional area of the nanocomposites. Therefore, if one assumed the material had the same conductivity, it would be expected that the measured conductivity of the sensors should increase as porosity decreased due to more initial electrical networks. However, this was not the case in Figure 3b, demonstrating that the material lost substantial conductive networks during the precuring process. A major contribution to this finding was a settling effect during the precuring duration that decreased the nanoparticle dispersion quality by reforming agglomerates due to gravity and van der Waals forces between the MWCNTs. In our previous work, we found that one-step microwave-curing a MWCNT doped elastomer likely increased the nanoparticle alignment within the fabricated nanocomposites, which resulted in significant electrical conductivity enhancements [37]. However, the alignment effects of microwave irradiation on MWCNTs within a resin likely decreased as the viscosity of the matrix increased due to precuring, consequently limiting MWCNT movement and alignment.

As the thermoset resin cured its viscosity increased, which inhibited the ability of dipoles within the resin to orient in the direction of the electromagnetic field, effectively decreasing heating from microwave irradiation [41]. This effect is also known to lead to a decreased dielectric loss factor due to partial curing of the resin. Notably, Fotiou et al. claimed to have negated this effect using MWCNTs dispersed in an epoxy matrix, but this effect had not yet been explored for an elastomer containing dispersed MWCNTs [36]. Although the exact mechanisms behind microwave heating of MWCNTs are not fully understood, it is argued that two main mechanisms lead to the heating of MWCNTs under microwave irradiation: Joule heating as a result of imperfections in the carbon nanotubes and the transformation of electromagnetic energy to mechanical vibrations [42]. These vibrations are believed to be eliminated when the nanotubes are dispersed in a dense and viscous environment [42]. Therefore, as the viscosity of the prepolymer is increased due to partial precuring at room temperature, the subsequent microwave exposure heated the resin less effectively. The decrease in heating led to longer curing times, lower thermal gradients, and less THF evaporation before polymerization, which resulted in progressively higher density samples.

To obtain the nanocomposites with best piezoresistive sensing capability, a precuring time of one hour was determined to be most suitable for fabricating the porous nanocomposites. This precuring time was chosen to benefit from the uniformly distributed small pores for sensing and to keep the method time-efficient, while allowing for ample time to fabricate consistent density samples. This short waiting time proved to result in relatively consistent densities as the average density of the 16 fabricated samples was 0.93 ± 0.027 g/cm^3^.

### 3.3. SEM Investigation

**Figure 4:**
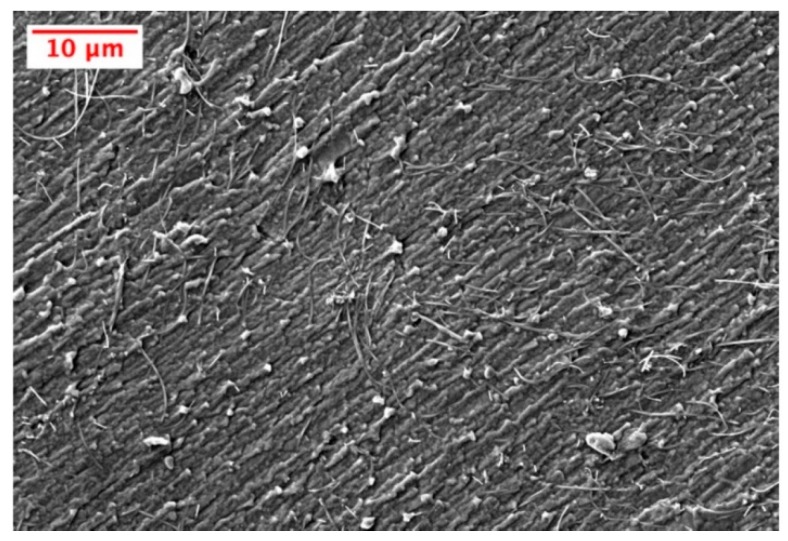
Representative SEM image of the quality of MWCNT dispersion in the polydimethylsiloxane (PDMS) matrix for a 2.5 wt. % sample.

Quality dispersion was important for reliable and consistent microwave-curing, electrical conductivity, and piezoresistive performance of the MWCNT doped elastomer. The cross-section of a 2.5 wt. % sample was imaged via SEM to investigate the dispersion quality. A representative image showing the quality of MWCNT dispersion is shown in Figure 4.

During the SEM imaging process, no agglomerates were identified. When exposed to microwave irradiation, the well-dispersed MWCNTs in the thermoset resin rapidly heated the prepolymer internally, leading to a tremendously time-efficient curing procedure. Agglomerates of MWCNTs are known to superheat under microwave exposure due to their high MWCNT concentration and elevated microwave absorption, which can lead to virtually immediate polymerization near the aggregate or potentially localized thermal degradation of the polymer [36]. Quality dispersion was also beneficial to the electrical and piezoresistive performance of the nanocomposites as higher dispersion means MWCNTs were more evenly spaced to achieve the most conductive networks throughout the nanocomposite. Additionally, during mechanical deformation of the matrix material, isolated MWCNTs had the potential to reorganize and form new conductive networks, which resulted in a greater change in resistance due to quality dispersion.

**Figure 5:**
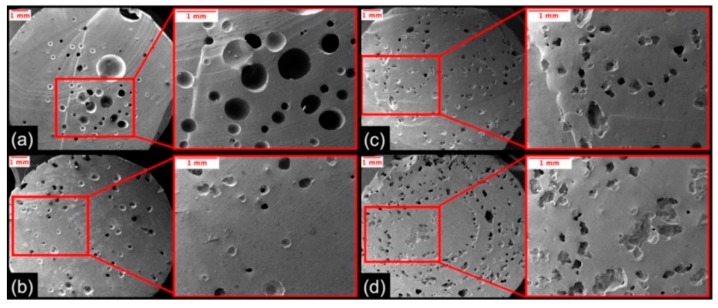
Cross-sectional SEM images of the porosity within nanocomposites containing MWCNT loadings of (**a**) 1.0 wt. %, (**b**) 1.5 wt. %, (**c**) 2.0 wt. %, and (**d**) 2.5 wt. %.

The size, shape, and distribution of the pores formed during microwave-curing impacted the mechanical and piezoresistive behavior of the nanocomposites. When the porous nanocomposite was compressed, the pores provided internal void space for the material to expand into. When the pores collapsed due to mechanical compression, new conductive networks were formed, which further decreased the material’s measured resistance. The MWCNT loading was observed to affect the porosity of the microwave-cured nanocomposites. Therefore, it was necessary to characterize these pores via SEM to gather insights on how these pores were formed and how they may have impacted the properties of the samples. Cross-sections of the nanocomposites with MWCNT loadings of 1.0 wt. %–2.5 wt. % were imaged via SEM to examine their pore morphology and distribution. Low and high magnification SEM images of the porosity within the nanocomposites are shown in Figure 5.

Residual solvent evaporation and expansion, thermal gradients, and potentially some localized thermal degradation of the polymer due to microwave-curing were the three likely mechanisms that induced porosity in the nanocomposites. The differences in the size, shape, and distribution of these pores were dependent on the process by which the pores were formed. The large circular pores found in the 1.0 wt. % sample (Figure 5a) were likely the result of residual solvent evaporation and release from the sample. As the material was heated past the THF boiling point (66 °C) during the microwave-curing process, the evaporated THF released before the cross-linking of polymer chains had fully propagated. Small holes were observed on the top of only the 1.0 wt. % samples after microwave irradiation and before removal of the expanded material beyond the mold. This indicated that the relatively low viscosity of the nanocomposite prepolymer allowed some evaporated THF to propagate through the sample and release before being fully cured. In contrast, the nanocomposite resin with 1.5 wt. % loading or higher exhibited high enough viscosity that the evaporated THF was not able to propagate through the sample before rapid polymerization occurred. Notably, it was observed that the inner core of the conductive material cured first under microwave irradiation.

As MWCNT content increased, the pores progressively decreased in size, increased in number, improved in distribution, and became more amorphous. These changes in porosity may be due to a progressive shift of the dominant role of pore formation from solvent evaporation and release to a localized expansion of the evaporated solvent that was unable to propagate through the resin material before polymerization occurred. The higher viscosity nanocomposite resins prohibited the evaporated sacrificial solvent from propagating through the resin to form larger pores. From this investigation, we found that the 2.5 wt. % nanocomposite had the largest number and best distribution of small closed-cell pores likely due to having the highest viscosity of the nanocomposite prepolymers.

### 3.4. Mechanical Characterization

It is widely accepted that MWCNTs supply substantial reinforcement to polymers when dispersed well within the matrix. For elastomers, this generally results in an increased Young’s modulus due to increased MWCNT loadings [43]. However, porosity also played a significant role in the stiffness of elastomeric nanocomposites, as they expectedly encouraged a decrease in the compressive modulus. Therefore, the mechanical properties of the porous nanocomposites needed to be characterized to determine the compressibility of each sample.

**Figure 6:**
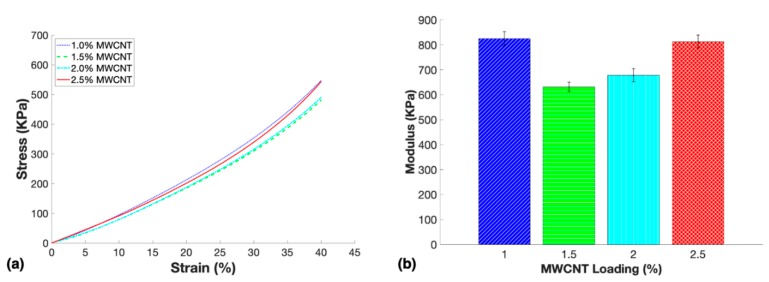
Mechanical characterization of the porous nanocomposites with loadings 1.0 wt. %–2.5 wt. % including: (**a**) stress vs. strain curves up to 40% compressive strain and (**b**) the average compressive modulus for each loading.

Figure 6a shows the stress–strain curves of samples at each MWCNT loading tested in this study. The stress–strain curve revealed a progressive increase of slope through the strain cycle indicating typical elastomeric behavior under compression. Thus, the compressive modulus of each sample was extracted from the most linear portion of the curve (between 0.1%–0.6% strain). The stress–strain curves in Figure 6a for 1.5 wt. % and 2.0 wt. % samples aligned well throughout the 40% strain cycle, considering they have similar porosities, Young’s modulus, and MWCNT reinforcement. In contrast, 1.0 wt. % and 2.5 wt. % had very similar Young’s modulus, but the slope of the two curves throughout the 40% strain cycle differed slightly which demonstrated the mechanical influence of the significant difference in porosity and MWCNT reinforcement. The stress–strain curves shown in Figure 6a aligned well with the average compressive modulus results shown in Figure 6b such that 1.0 wt. % and 2.5 wt. % samples were similar, and 1.5 wt. % and 2.0 wt. % samples were similar.

Surprisingly, nanocomposites with the lowest loading of 1.0 wt. % demonstrated the highest average compressive modulus of 824.8 KPa. This result may be explained by the small number of large pores in the 1.0 wt. % samples being located towards the top of the nanocomposites in the middle of the cross-section, leaving the majority of the sample completely solid to carry most of the load. Thus, the varying porosity of the nanocomposites played a significant role in the mechanical properties, as it has been previously proven that higher concentrations of carbon nanotubes increased the stiffness of viscoelastic material [43]. The compressive modulus of the 1.5 wt. %, 2.0 wt. %, and 2.5 wt. % samples increased with higher loadings of MWCNTs as expected, indicating that the differences in the pore structure between these samples did not affect the modulus of the nanocomposites more so than the increase in MWCNT reinforcement.

### 3.5. Electrical and Piezoresistive Characterization

**Figure 7:**
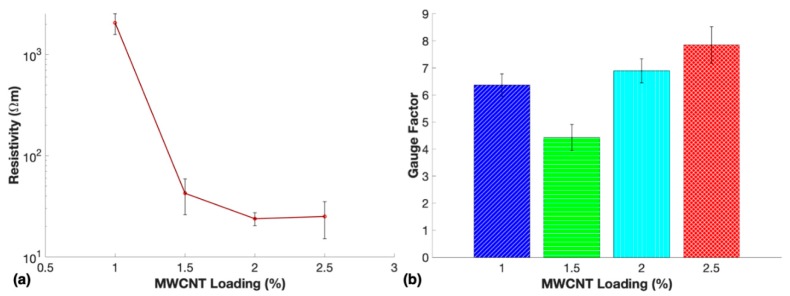
(**a**) Resistivity measurements and (**b**) average gauge factors at 10% compressive strain of porous nanocomposites containing 1.0 wt. %, 1.5 wt. %, 2.0 wt. %, and 2.5 wt. % MWCNTs.

The resistivities of each sample were measured to investigate the number of conductive networks within the nanocomposites that contained various loadings of MWCNTs (Figure 7a). Resistivity measurements of the nanocomposites confirmed an expected decrease in resistivity due to increased MWCNT loading. Samples fabricated with loadings less than 1.0 wt. % were determined to be nonconductive with our testing setup, therefore, they were not suitable for this study. Interestingly, the average resistivity of 2.5 wt. % nanocomposites was slightly higher than the average resistivity of 2.0 wt. %. However, both averages were past the percolation curve and within the error bars of the other, which indicated that further loading of MWCNTs would only exhibit a modest change in electrical properties.

Normalized (relative) resistance change and gauge factor were used to evaluate the piezoresistive sensing performance of the nanocomposites under mechanical deformation including the relative resistance change was calculated using Equation (1):

(1) $$Relative\;Resistance\;Change=\;\frac{R-R_{o}}{R_{o}}\times 100\%\;=\;\frac{\Delta R}{R_{o}}\times 100\%$$

where *R* is the immediate measured electrical resistance and *R_o_* is the initial resistance before strain was applied. The gauge factor is the normalized resistance change at a particular strain and was calculated using Equation (2):

(2) Gauge Factor=  R−RoRoε= ΔRRoε

where *ε* is the applied strain on the nanocomposite. These metrics were calculated based on the measured resistance data collected during cyclic compression tests to measure the piezoresistive performance of each sample.

The first sensing test to determine the optimal MWCNT loading of the nanocomposite sensors was a cyclic loading test at 10% strain to determine which loading had the highest average gauge factor. Gauge factors for the microwave-cured nanocomposites at loadings of 1.0 wt. %–2.5 wt. % are shown in Figure 7b.

Typical gauge factors for bulk piezoresistive sensors tend to increase with lower conductive filler content due to the higher resistivities allowing for a larger change in relative resistance upon deformation. This is due to the lower conductivity material having more incomplete conductive networks that more often form new complete electrical pathways upon deformation compared to the saturated networks within higher loading piezoresistive sensors. However, this is not the case in the present study likely due to the piezoresistive effects caused by the pores that collapsed upon compression in each sample. Under 10% compressive strain, the highest average gauge factor was 7.9 for the 2.5 wt. % nanocomposite. The results shown in Figure 7b suggest that the highest number of small pores well-distributed throughout the samples produced sensors with the highest sensitivity. One mechanism that may have contributed to the increased sensitivity of the more porous samples was the reduction in the number of initial conductive pathways thus leading to increased sensitivity [44]. It has been reported that porous PDMS carbon nanofiber sensors did not exhibit the best sensitivity for the lowest conductivity samples as would be expected for bulk material [12,45]. This may be attributed to the increased contribution of resistance change from collapsed pores of higher conductivity material forming a higher number of new conductive networks than lower conductivity material. Therefore, the collapsed pores may have been a larger contributor to the relative resistance change upon compression than the reorganization of MWCNTs within the matrix material. The nanocomposites with 2.5 wt. % MWCNTs displayed the highest sensitivity, therefore further studies were performed to fully characterize their piezoresistive performance.

**Figure 8:**
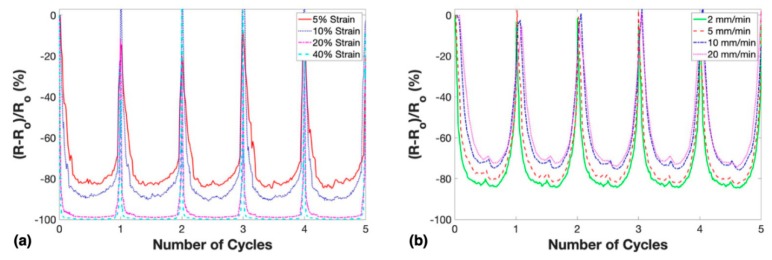
Resistance change as a response to cyclic testing of porous nanocomposites with 2.5 wt. % MWCNTs: (**a**) at various maximum strains and constant crosshead speed and (**b**) at various loading rate and constant maximum strain.

To further study the versatility of these compression sensors, a 2.5 wt. % nanocomposite was subjected to cyclic loading at maximum strains of 5%–40% at a constant crosshead speed of 2 mm/min and cyclic loading at crosshead speeds of 2–20 mm/min at a constant maximum strain of 10%. The piezoresistive response of the tests varying maximum strain and loading rates are shown in Figure 8.

There are two widely recognized mechanisms behind the change in resistance due to compression for bulk MWCNT-based nanocomposites. First, the decrease in length and increase in the cross-sectional area of the conductive material will decrease measured resistance due to compressive strain. Second, the carbon nanotubes dispersed within the matrix material reorganize to create new and destroy old conductive networks. During compression, more electrical networks are formed than are destroyed due to the carbon nanotubes generally being brought closer together, decreasing the average tunneling distance and creating new MWCNT–MWCNT contacts. Notably, tunneling resistance is widely recognized as the dominant mechanism that influenced the electrically conductive networks within MWCNT-based nanocomposites [46]. The normalized resistance decreased upon compressive loading and increased during unloading, therefore indicating negative piezoresistive behavior.

Porosity contributed to additional resistance change for the microwave-cured nanocomposites under compressive loads. Upon compression, the pores began to collapse as they were compressed in the longitudinal direction of the applied load and were stretched in the lateral direction due to a high Poisson ratio of the matrix material. PDMS has a very high Poisson ratio (between 0.45–0.5) which led to the small closed-cell pores in the 2.5 wt. % nanocomposites to collapse fully at smaller strains than an elastomer with lower Poisson ratio [47]. As a closed-cell pore collapsed, new electrical networks were formed which further decreased the resistance during compressive strain.

The gauge factors of the best 2.5 wt. % porous nanocomposite at strains of 5%, 10%, 20%, and 40% strains were 16.6, 9.2, 4.9, and 2.5 respectively. This approximately linear decrease was expected as the strain increased. These gauge factors were similar to what has been reported for bulk MWCNT-based flexible compression sensors in the past, which demonstrated that microwave-curing could be used as a time-efficient method to fabricate these nanocomposite sensors [8]. As the nanocomposite was compressed and percolating networks were completed, the potential for completing more networks decreased as the pores collapsed completely and the electrical pathways in the material became saturated. A small hysteresis effect was apparent at 5% and 10% maximum applied strains where at maximum compression the resistance rose slightly at the point of highest strain. This behavior has been previously observed in literature and although it is not well understood, researchers have claimed that it is likely due to the competition between network formation in the longitudinal direction of the applied compressive load and network breakdown due to a high Poisson ratio or conductive filler damage [14,48]. Interestingly, at 20% and 40% strains, no hysteresis effect was revealed, and the minimum relative resistance remained very stable throughout the compression cycle.

The results for testing the best 2.5 wt. % porous nanocomposite at varying loading rates indicated that the relative resistance change decreased as the loading rate increased. The difference of minimum normalized resistance between crosshead speeds of 2 and 20 mm/min was 10.3%. This phenomenon could be explained by the collapsed pores within the conductive nanocomposite had progressively less time to reach equilibrium upon faster applied cyclic loads, therefore, conductive networks that were completed at slower crosshead speeds were not completed at faster crosshead speeds. Notably, most of the decrease in relative resistance change was between 5 and 10 mm/min, which signified that this performance displayed asymptotic behavior at higher crosshead speeds. In a compression sensing application with known strain, this porous nanocomposite could be used to not only measure the applied strain but measure the loading rate as well.

**Figure 9:**
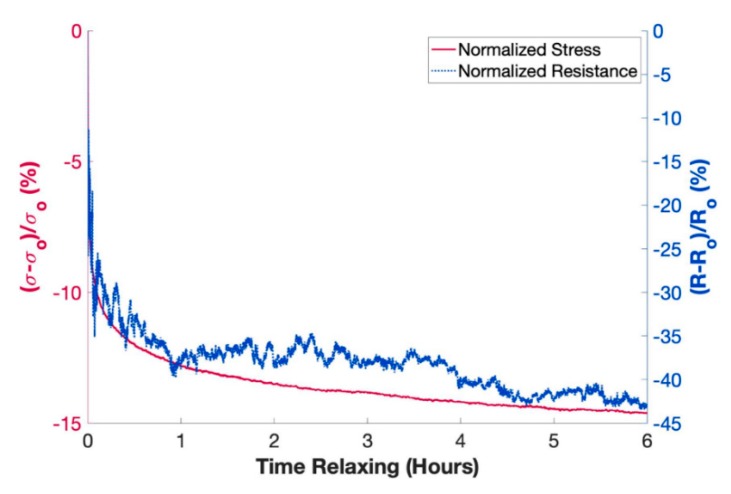
Piezoresistive change during stress relaxation behavior of a 2.5 wt. % porous nanocomposite held at 10% compressive strain for 6 h.

The long-term piezoresistive and stress relaxation behavior of the nanocomposite held under compressive strain was characterized. The 2.5 wt. % microwave-cured nanocomposite was compressed to a maximum strain of 10% and held for 6 h while recording mechanical and electrical data. The results of this test are shown in Figure 9. Notably, the stress and resistance signals shown depict the data recorded after 10% strain was applied. The results indicated that the majority of the piezoresistive and stress relaxation behavior demonstrated by the nanocomposite took place within the first hour. The measured rate of stress relaxation agreed well with the reduction of the measured resistance change of the nanocomposite. This confirmed that the electrical networks formed by the MWCNTs within the PDMS matrix were dependent on the viscoelastic stress relaxation behavior of the matrix material. Small variations in the relative resistance change can be attributed to the saturation of the percolation networks of the 2.5 wt. % nanocomposite upon compression. Over a few hours, these electrical networks became progressively more stable indicative of increased stress stability within the matrix material.

### 3.6. Sensing Application

**Figure 10:**
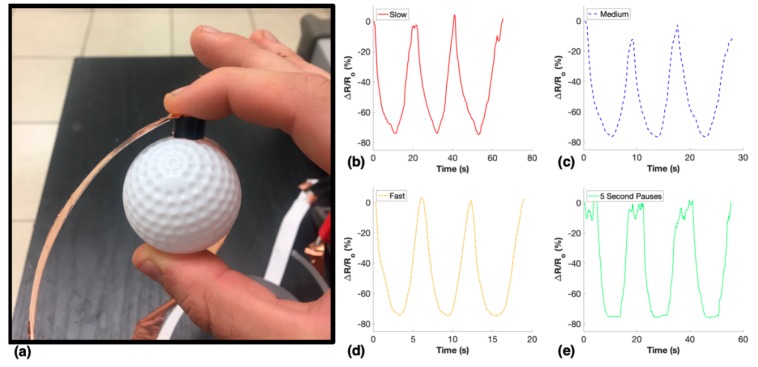
Sensing application of 2.5 wt. % porous nanocomposite: (**a**) experimental setup and sensing functions during various squeezing rates including: (**b**) slow, (**c**) medium, (**d**) fast, and (**e**) 5 s pauses at maximum and minimum compression.

The 2.5 wt. % porous nanocomposite was used in a compression sensing application to prove is viability as a flexible sensor that could be used in a variety of applications including skin attachable human motion detection, soft robotics, and prostheses. The piezoresistive sensor was placed between two copper tape electrodes and the resistance of the nanocomposite was measured while compression was applied by squeezing a golf ball, as seen in Figure 10a. The golf ball was cyclically squeezed at four different rates and the relative change of resistance of the nanocomposite for each rate is shown in Figure 10b–e. Notably, the sensitivity of the sensor remained consistent throughout each test and did not display dependence of strain rate as the maximum strains and strain rates applied were unknown.

## 4. Conclusions

In this paper, a facile microwave-curing method was used for rapid fabrication of porous PDMS matrix nanocomposites containing dispersed MWCNTs. This rapid fabrication method produced piezoresistive sensors in under 30 s, which led to significant time savings compared to the nanocomposites cured by traditional thermal-curing methods. The effects of partial precuring of the nanocomposite at room temperature before microwave-curing were examined to determine the best procedure for manufacturing consistent piezoresistive sensors with beneficial closed-cell and well-dispersed porosity. Dispersion quality of MWCNTs in PDMS elastomer was confirmed using SEM images. The increased loadings of MWCNTs induced a larger number of small closed-cell pores within the nanocomposites due to residual solvent evaporation and expansion not propagating through the high viscosity prepolymer during the microwave-curing procedure. Experimental results showed that the nanocomposite with 2.5 wt. % MWCNTs had the highest piezoresistive sensitivity due to the improved microporous structures and can be effectively used as a compression sensor involving large deformations. The piezoresistive properties and mechanical properties of the porous nanocomposites compression sensors were characterized to verify their stable performance over multiple cycles and loading rates. Finally, the 2.5 wt. % nanocomposite was demonstrated as a viable fingertip sensor when cyclically squeezing a golf ball at different rates.
